# Mismatched transfusion of 8 AB0-incompatible units of packed red blood cells in a patient with acute intermittent porphyria

**DOI:** 10.4103/1658-354X.76497

**Published:** 2011

**Authors:** Burkard Rudlof, Burkhard Just, Robert Deitenbeck, Thomas Ehmann

**Affiliations:** *Klinikverbund St. Antonius und St. Josef Wuppertal, Institute for Anesthesia*; 1 *Blood Service of the German Red Cross Centre for Transfusion Medicine Hagen*; 2 *Department of General Surgery, Johanniter Krankenhaus Radevormwald*

**Keywords:** *Incompatible transfusion*, *porphyria*, *mixed field reaction*, *molecular genetic diagnostic*

## Abstract

We report on a patient with acute intermittent porphyria, who received 8 AB0 incompatible units of packed red blood cells in an emergency situation. She never showed any signs of severe intravascular haemolysis. The patient died after four weeks because of a multi-organ failure caused from the malpractice of the porphyria. The problems of bedside testing, mixing field reaction, fresh frozen plasma and molecular-genetic determination of bloodgroup were discussed.

## INTRODUCTION

Mismatched transfusions in the ABO-system entail more or less severe intravascular hemolysis, in some cases even combined with multiorgan failure and death. This is due to severe antibody reactions between circulating allo-antibodies and the corresponding antigens of the mismatched red blood cells (RBC). There are, however, a few case reports on the absence of hemolysis despite major incompatibility.

An intensive literature search revealed only 18 reports on mismatched transfusions in the last 50 years.[1–14] A forensic search in Germany showed over 100 such occurrences in a 3 year period. Supposed that only 50% of these cases lead to a complaint, this means five clinically relevant mismatches per 100,000 transfusions. This also corresponds roughly to the numbers published by Linden *et al*.[15] in New York with 5.2 per 100,000 where a strict mandatory reporting system was established. Our assumption, that reports on mismatched transfusions are frequently suppressed because of the forensic consequences which can be expected, has proven to be true, since a mismatched transfusion is usually the result of a severe and avoidable error by the physician. This is not beneficial neither to the calculation of risks nor to the examination of the therapies.

We consider it of utmost importance to report on this recorded unusual case, its course, and the medical consequences.

## CASE REPORT

A 64–year-old female patient was admitted to a medical department because of unclear upper abdominal complaints. An upper gastroscopy and an ERCP proved to be normal. Two hours after an unremarkable colonoscopy the patient’s condition deteriorated clinically, and her hemoglobin dropped from 11.6 to 8.1 g/dl. In the next 4 h the patient’s state worsened so much that she had to be admitted to an intensive care unit. In the first red blood count on the intensive care unit, the patient’s hemoglobin was 2.7 g/dl. A fast ultrasound test of the abdomen showed free fluid in the abdomen with a strong suspicion of a splenic rupture. At the same time two specimen for compatibility testing were sent to the laboratory. In the laboratory two serum tubes marked with electronically generated labels with the name of the patient arrived. The on-duty medical laboratory technician determined the blood group from a tube, performed an antibody screening and compatibility testing. The blood group was determined to be A (D positive). Two A (D positive) units of packed RBC were transfused immediately without bedside testing because of the patient’s critical condition.

In the operating room she received another 4 units of packed RBC of blood group A (D positive). A bedside test before transfusion of these units now showed a blood group A. The transfusing anesthetist had no reason to doubt the result of the laboratory or the bedside test. Six units of fresh frozen plasma of blood group A were administered. After a splenectomy the patient was transferred to the intensive care unit with stable circulatory conditions and artificially ventilated, where she was again given 2 units of packed RBC of blood group A (D positive).

The next morning a double check of blood group took place via 2^nd^ medical laboratory technician from the 2^nd^ Tube. The result was blood group O (D positive). Thus the blood group was determined again from the 1st Tube, and the result A (D positive) was confirmed. A further blood withdrawal from the patient resulted in the blood group of A (D positive) without mixing field reaction. Now there was need for clarification whether this now was the patient’s genuine blood group or the blood group of the units of packed RBC after a complete blood exchange.

In order to determine unequivocally the woman’s blood group, two molecular-genetic diagnostics procedures were used. They clearly determined blood group O. Moreover, one earlier result of blood group testing by another hospital was found, which also positively confirmed blood group O (D positive). Therefore, the first sample must have come from another patient and had been marked incorrectly.

Since hemolysis had to be expected, a forced diuresis was introduced immediately and hemolysis parameters in the serum were tested regularly. At no time, a clinically perceptible intravascular hemolysis occurred. In addition, the serum parameters and the anti-A titers rose only slowly. However the hemoglobin dropped slowly to the transfusion limit, without further blood loss. This must be probably regarded as indication of an extravascular hemolysis [Table 1].

**Table 1:** Hemolysis parameters, anti A titer and A antigen

Day	0	1	2	3	4	5	6	7	8	9	10	11	12	13
HB (g/dl)	11.6	9.6	7.6	7.2+	11.2	9.6	9.5	9.0	8.3	7.1	10.6+	9.5	10.3	10.1
LDH (U/l)	210	334	257	252	212	232	230	424	300	286	252	244	191	328
Bilirubin (mg/dl)	0.67	0.77	0.46	0.32	0.24	0.45	1.25	1.67	1.43	0.64	0.75	0.68	0.77	0.85
Anti-A titer		0	0	1	1		2	4	16	64	128			
A-Antigen		A	A	A(0)	A(0)	A(0)	0(A)	0(A)	0(A)	0(A)	0	0	0	0

Day 0=day prior to transfusion, Day 1=day of the transfusion; +=2 units of RBC

After 3 days an acute intermittent porphyria was found as a secondary finding; this was probably the cause of the unclear abdominal complaints. The therapy was therefore adjusted to trigger-free medication.

The further intensive therapy process was first characterized by repeated generalized convulsions and later by a progressive multi-organ failure (MOF), which most probably can be explained by the initially inappropriate therapy of the porphyria. The patient deceased 4 weeks after the event because of a multi-organ failure (MOF) without ever showing signs of severe intravascular hemolysis.

## DISCUSSION

### Incorrect marking of the tubes

In total 58% of errors resulting in the administration of incorrect blood are caused by failure to identify the patient, errors during withdrawal of blood, wrong patient’s name and emergency situations.[16] Only 25% of the cases are owing to errors in the blood bank.

Most recipients of ABO incompatible blood have blood group O[17] as in our case.

Thus, it seems that the present case study is very typical for a transfusion of incompatible blood.

We believe that the safety of blood with regard to infections makes the user think that all aspects of blood transfusions are safe. Since infectious complications have been reduced, the main reason of fatal transfusion complications is the transfusion of mismatched blood.

### Bedside test

**Figure 1:**
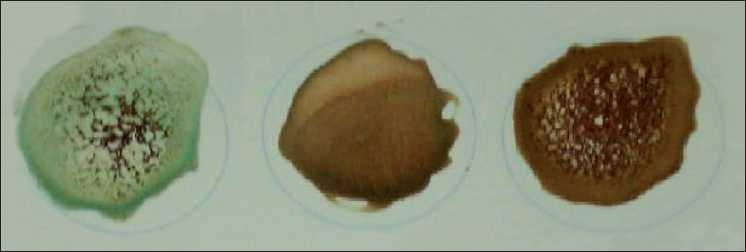
Left side: anti- A with A blood; Middle: anti-A with O blood; Right side: anti-A with A- and O blood (= mixing field reaction)

A bedside test carried out prior to administration would have prevented the mismatched transfusion. Situations that do not allow bedside testing are difficult to imagine, because there should be enough time to do the test while the packed RBC are delivered from the blood bank. If blood-group-unequal blood has already been given, it is very hard to determine the correct blood group. In the aforementioned case the blood group in the bedside test was determined incorrectly, since first only the agglutination became evident. It can not be determined afterward, but it is to be assumed with large probability that a mixing field reaction did take place. Recognition of a mixing field reaction in the bedside test is nearly impossible for doctors, who do not have cross sample laboratory experience [Figure 1].

### Fresh frozen plasmas

The administration of the fresh frozen plasma (FFP’s) of blood group A can be regarded as unproblematic since no anti-bodies to the recipient’s RBC are present. This practice can be adhered to even in the case of supply.

In the present case it could have been advantageous if the patient had not received further A-antibodies. We think that, after a mismatched transfusion, patients should receive only FFP’s without antibodies to transfused or recipient’s RBC. In case of doubt AB-FFP’s are free of antibodies.

### Molecular-genetic determination blood group

In the majority of cases the immune hematological determination of the blood group is sufficient and safe. However, after mass transfusions and polytransfusions (e.g. homozygote thalassemia), it may be difficult to differentiate between the group of the donor’s and the patient’s blood group. The molecular-genetic blood group determination offers a solution in such a case. Here it would have otherwise been impossible to determine the real blood group of the patient, since even the old test result of blood group could have been based on a mix-up of samples.

### Missing of intravascular hemolysis

The authors have already been involved in the treatment of several ABO incompatible transfusions. However, these cases involved transfusions of up to2 units. Without exception severe circulatory reactions and indications of an intravascular hemolysis occurred. Among them were also acutely lethal processes.

Initially, it appears to be rather amazing that the mismatched transfusion of 8 units should lead to obviously substantial milder reactions. As a consequence of this extraordinary case, we contacted several German transfusion centers and heard about several similar cases with up to 20 mismatched units, which remained without any acute effects just like the case described above.

After literature research, it seems obvious that there is no correlation between the number of the transfused units and the type and severity of the reaction. Fatal outcomes are described after 1, 4, and 30 units.[2,6,8] Lethal processes without direct connection with the transfusion occurred after 9 and 15 units.[2,12] After 4×1, 2, 2×3, and 4 units[1,2,4,6,7,11,13,14] it came to serious complications, and without consequences remained the mismatched transfusion of 3×1, 3, and 7 units.[2,3,9–11] This represents a rate of 5.3% with fatal outcome. Linden *et al*.[16] found a comparable rate of 3% in a prospective study.

Yang *et al*.[18] report on two cases of consciously mismatched transfusion during liver transplantation, because it was not possible to supply the O-recipients with blood group O red blood cells (RBC). These case studies occurred at least 20 years ago, when a large loss of blood during liver transplantation was normal. The first 10 units were given as O-RBCs. Parallel fresh frozen plasma (FFP) was given to dilute the Anti-A-titers. The main blood loss was managed with A-RBCs. During the last stage of surgery the patients again received O-RBCs. No hemolysis occurred even after receiving 40 or 93 units of A-RBCs, respectively.

These case studies describe nearly the same procedure in another dimension. The only difference was that the patients received cyclosporine and methylprednisolone after liver transplantation for immunosuppression. Whether this therapy is useful for the prevention of a hemolysis is very questionable. In the acute process the lack of stronger reactions can be explained by the complete loss of the patient’s own plasma and the antibody caused by the severe bleeding. The following new formation of the anti-bodies obviously results in only a slow increase in the titers, so that an acute reaction is absent. In order to monitor risks and possible therapies more effectively, it is necessary to develop an at least national mandatory reporting system, which should exclude each forensic access. A standardized questionnaire should collect information about the type of mismatched transfusion, the transfused quantity, the used therapies and the outcomes.
